# Mortality, fertility, and economic development: An analysis of 201 countries from 1960 to 2015

**DOI:** 10.12688/gatesopenres.12804.1

**Published:** 2018-03-01

**Authors:** Qingfeng Li, Amy O. Tsui, Li Liu, Saifuddin Ahmed

**Affiliations:** 1 Bill & Melinda Gates Institute for Population and Reproductive Health Department of Population, Family and Reproductive Health, Johns Hopkins Bloomberg School of Public Health, Baltimore, MD, 21205, USA

**Keywords:** Mortality, fertility decline, demographic dividend

## Abstract

**Background:** The efficient utilization of the economic opportunities effected by rapid reductions in fertility and mortality is known as the demographic dividend. In this paper, our objectives are to (1) estimate the contribution of fertility and mortality decline during the period 1960-2015 to demographic dividend due to change in age structure, and (2) assess the economic consequences of population age structure change.

**Methods** **:** Employing the cohort component method, we performed population projections with different scenarios of changes in mortality and fertility between 1960 and 2015 in 201 countries. We specifically focused on low- and middle-income countries in Asia, Latin America and the Caribbean (LAC), Northern Africa, and sub-Sahara Africa (SSA)

**Results:** The child dependency ratio, defined as the number of children (0-14 years) per 100 working age population (15-64 years), would be 54 higher than the observed level in 2015 in both Asia and LAC, had fertility not declined. That means that every 100 working age population would need to support an additional 54 children. Due to the less substantial fertility decline, child dependency ratio would only be 16 higher if there were no fertility decline in SSA. Global GDP (constant 2011 international $) would be $19,016 billion less than the actual level in 2015 had the fertility decline during 1960-2015 not occurred, while the respective regional decreases are $12,390 billion in Asia, $1,985 billion in LAC, $484 billion in Northern Africa, and $321 billion in SSA.

**Conclusions:** SSA countries may accelerate the catch-up process in reducing fertility by investing more in family planning programs. This will lead to a more favorable dependency ratio and consequently facilitate a demographic dividend opportunity in SSA, which, if properly utilized, will spur economic development for the coming decades.

## Introduction

Rapid reductions in fertility and mortality during the last half-century have resulted in dramatic changes to the population age structures of many countries, which economists have argued have been demonstrably conducive to economic development ^1^. Countries with a high proportion of their population in working ages are better able to use their resources for economic development due to reduced expenditures related to caring for child and elderly dependents. The efficient utilization of the economic opportunities that result in part from a favorable demographic transition is termed “the demographic dividend”.

The association between fertility and mortality declines and the demographic transition has been extensively studied and well documented, but their relationship with economic development has not been systematically investigated in the health literature ^2^. A comprehensive demonstration of its health and economic benefits strengthens the advocacy for fertility decline through programs that directly influence fertility levels, such as meeting women’s need for family planning.

Such an investigation links several sustainable development goals (SDGs). Fertility decline results in a smaller total population, which alleviates the burden on earth’s life-support system imposed by a global population set to rise to 9 billion by 2050 ^3^. Women empowered to adapt voluntary measures to reduce fertility will benefit themselves, their children, and the local and global economy and environment ^4^.

Over two centuries ago, Malthus argued that unconstrained population growth would lead to catastrophic consequences because the amount of many production factors, such as land, is fixed ^5^. Solow subsequently proposed that even reproducible factors would be swamped by rapid population growth ^6^. The variation in population growth rates is an important factor in explaining differences in long-term economic performance across countries. The implications of the theories proposed by Malthus and Solow, as well as others, are pessimistic for countries with sustained high fertility rates. According to this framework, fertility decline results in a smaller total population, which in turn increases the ratio of fixed and reproducible factors to labor.

Additionally, lower fertility levels are also associated with higher investments in human capital, another important production factor ^7, 8^. Moreover, lower fertility means that women’s time spent on bearing and caring for children declines and may translate into a higher female labor participation rate, which independently contributes to the economy ^9^.

At the aggregate level, fertility also significantly impacts the population age structure. Lower fertility implies fewer children and a lower child dependency ratio, defined as the ratio of children (i.e. aged 0–14 years) to the working age population (i.e. aged 15–64 years). Holding other factors constant, such as the labor participation rate, a larger proportion of working age population can lead to greater output per capita. 

**Figure 1.**
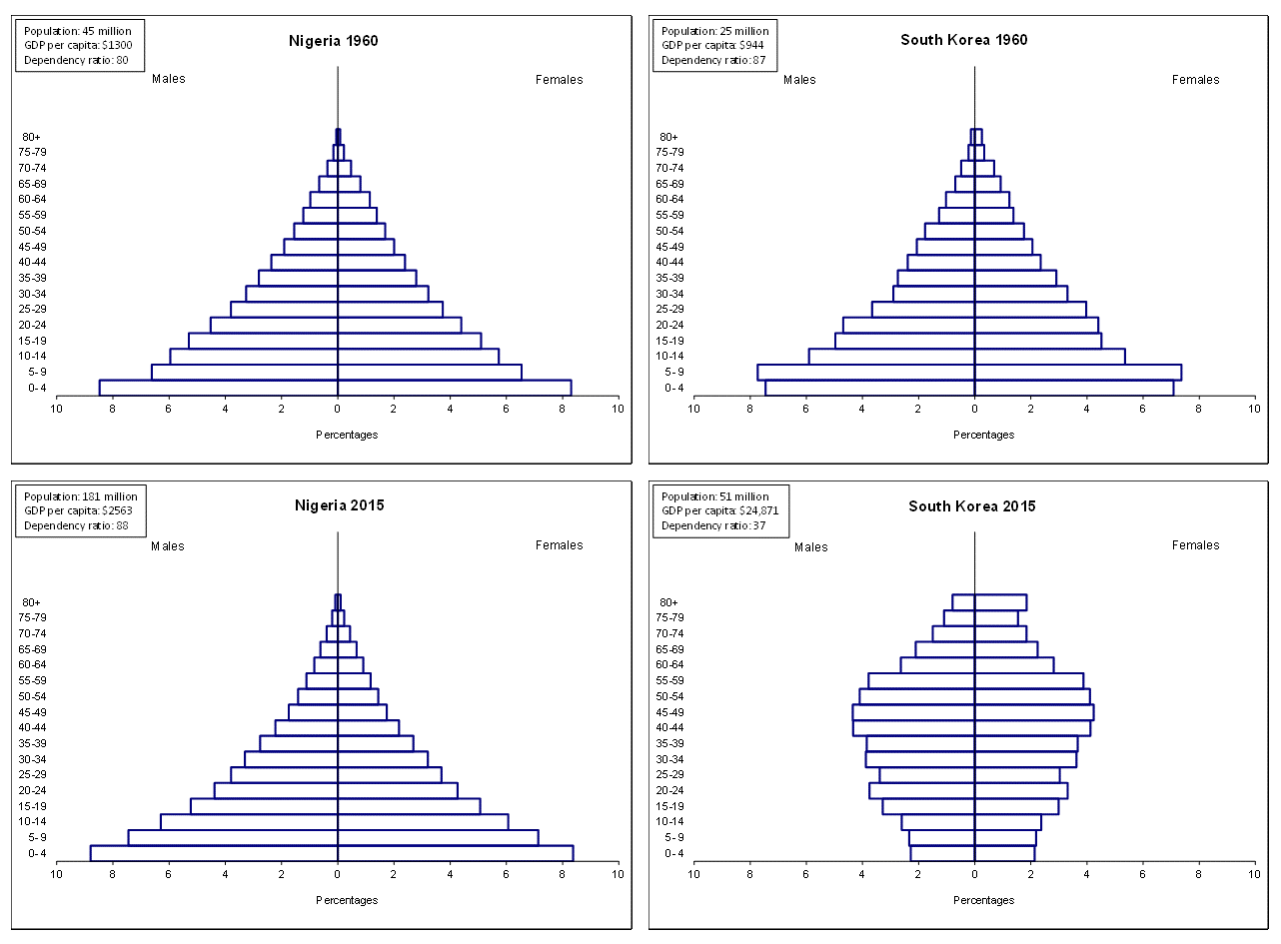
Population pyramids of Nigeria and South Korea in 1960 and 2015.

Empirical studies have identified a strong correlation between a favorable population age structure and rapid economic growth ^10, 11^. It has been estimated that as much as one-third of the economic growth in the “East Asia Miracles” economies of Hong Kong, Singapore, South Korea, and Taiwan, from the early 1960s to 1990s, was derived from their rapid fertility transitions ^1^. Figure 1 compares the population pyramids of Nigeria and South Korea. In 1960 their population age structures were similar, with the dependency ratio (population aged 0–14 and 65+ years divided by the population aged 15–64 years) being 80 and 87 in Nigeria and South Korea, respectively. According the World Bank, the GDP per capita (constant 2011 US$) was 50% higher in Nigeria than South Korea in 1960. Fifty-five years later, the dependency ratio had decreased to 37 in South Korea while it increased to 88 in Nigeria. During the same period, the GDP per capita rose to $24,871 in South Korea, a 26-fold increase. The increase was less than 2-fold in Nigeria in constant dollars.

**Figure 2.**
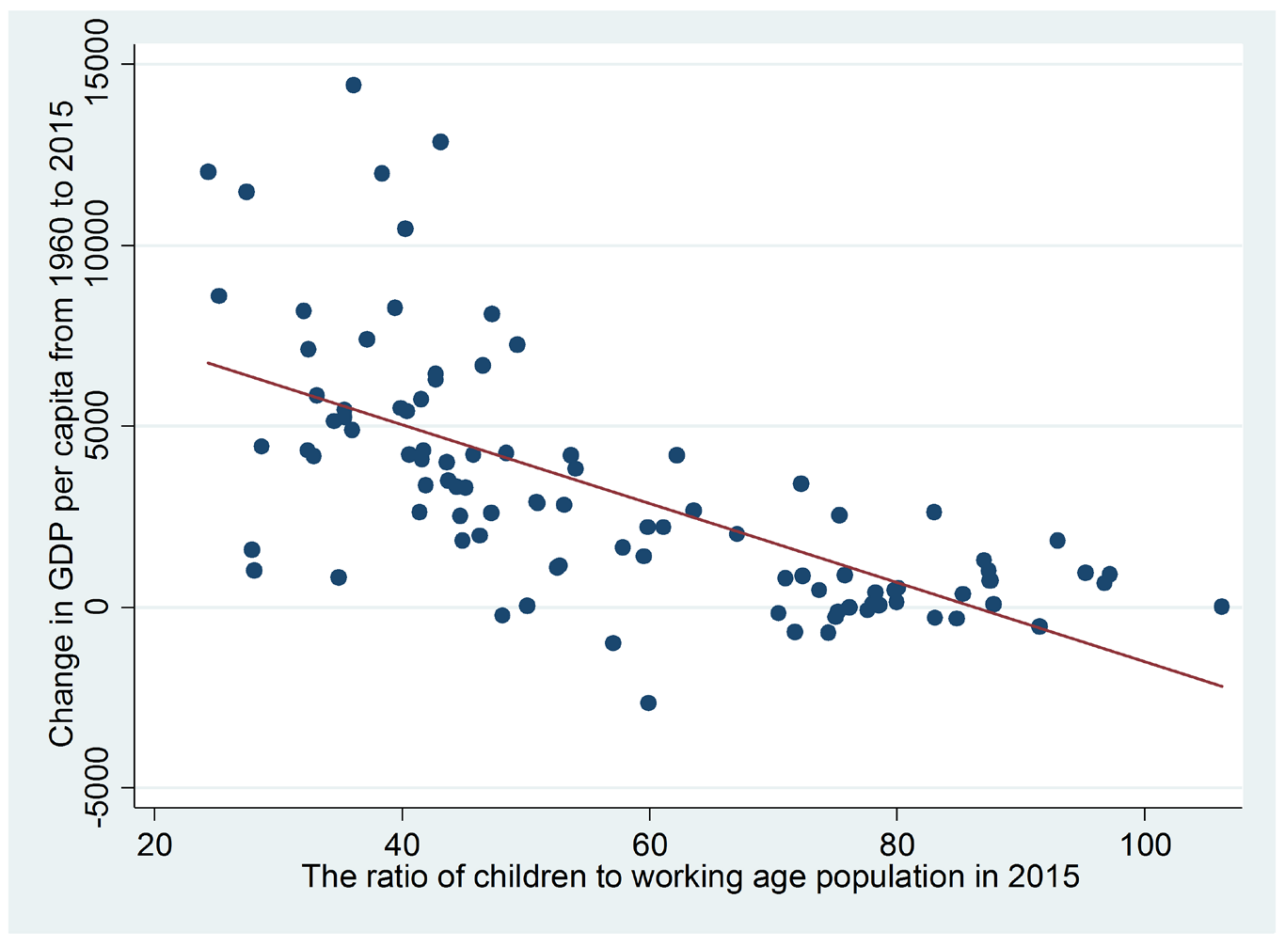
Change in GDP per capita from 1960 to 2015 and the ratio of children to working age population in 2015 in 120 low- and middle-income countries in Asia, LAC, Northern Africa, and SSA.

Figure 2 illustrates the relationship between economic growth and the ratio of children to working age population in 120 low- and middle-income countries (LMICs) in Asia, Latin America and the Caribbean (LAC), Northern Africa, and sub-Sahara Africa (SSA). The vertical axis is the change in GDP per capita during the period 1990 to 2015. GDP is based on purchasing power parity (PPP) and is measured in constant 2011 international dollars. The horizontal axis is the child dependency ratio in 2015. We use 1990 as the starting year, instead of 1960 that is used in subsequent sections, since PPP-converted GDP data only first became available in the World Bank database in 1990. High-income countries are excluded since most of them had completed their demographic transitions long before 1990. The inverse relationship between economic growth and the ratio of youth to working age population during the past two decades is consistent with findings from previous studies ^12^. This helps justify our use of the child dependency ratio as the indicator with which to investigate the relationship between mortality, fertility, and economic development. During the 25-year period considered, the increase in GDP per capita was greater in those countries that had achieved a lower child dependency ratio by 2015. A linear regression analysis of the change in GDP per capita against the 2015 child dependency ratio shows a satisfactory goodness of fit with *R* ^2^ = 0.44. The slope of the linear fitted line is -109 (95% CI: -134,-85), In other words, each one- unit change in 2015 child dependency ratio is associated with 109 fewer international dollars in 2015 GDP per capita.

## Methods

### Data sources

We obtained data on the fertility, mortality, and population age structure for 201 countries during the decades 1960 to 2015 from World Population Prospects (WPP), the 2017 Revision. This is the 25 ^th^ round of official United Nations (UN) population estimates, published in June 2017 by the Population Division of the Department of Economic and Social Affairs of the United Nations Secretariat ^13^. The economic data were obtained from the International Comparison Program Database of the World Bank.

Dependency ratios are used as indicators of the population age structure. Similar to the child dependency ratio defined above, the aged dependency ratio is the ratio of the number of elders (65 years and above) to the working age population. The total dependency ratio equals the sum of child and aged dependency ratios. As a commonly-used fertility measure, total fertility rate (TFR) is the number of children a woman would have over her lifetime if she were to experience the observed period age-specific fertility rates.

### Cohort component method (CCM)

CCM is a demographic projection method used by the UN to generate WPP estimates. It employs a transition matrix to predict population by age from one period to the next. Following WPP 2017, our projections were made for five-year intervals. The basic equation for the CCM is

$$P_{t+5}=M_{t,t+5}*P_{t}\;(1)$$

where *P _t_* is a column vector whose elements are the age-specific population at calendar time *t*; *M _t,t_*_+5_ is a transition matrix constructed from the age-specific fertility and mortality.

All 201 countries included in the WPP 2017 database are used in this study. Among them, 187 countries have data on GDP per capita (PPP, constant 2011 international $) in the World Bank’s International Comparison Program Database. The majority of the figures and tables which follow below are based on countries in 4 regions (Asia, LAC, Northern Africa, and SSA) that are relevant to this study. Population projections for the following three scenarios were made using Stata 14: what would the 2015 dependency ratio be if, during the period from 1960 to 2015, there had been (1) neither a fertility nor mortality reduction; (2) no fertility reduction; (3) no mortality reduction? Based on these estimates we further assessed what the GDP per capita would be in 2015 under these three scenarios.

## Results

### Demographic implications of fertility and mortality declines

Globally, child and total dependency ratios declined significantly from 1960 to 2015, with large country-level and regional variations. The declines in SSA are the smallest among the five regions - the median change in both child and total dependency ratios is close to zero. On the other hand, Asia and LAC have experienced dramatic changes in both fertility levels and dependency ratios, with median changes in the range of 30 to 40 units.

The decompositions of the contributions from fertility and mortality declines to the change in dependency ratios were conducted at both regional and country levels. Table 1 and Table 2 show the results from country-level and regional-decomposition, respectively. Mortality decline was considered in the estimations since it is an important determinant of population age structure. However, our discussion is mainly on fertility for two reasons. First, as illustrated in Table 1 and Table 2, the effect of mortality decline is smaller than that of fertility change. Second, no government, including those facing a tremendous challenge of population aging, have ever proposed slowing mortality decline to make the population age structure more conducive to economic development ^14^.

The contribution of fertility decline to the change in the dependency ratio was smallest in SSA than in any other region. The 2015 child dependency ratio was 80 in SSA and would have been 96 had there been no fertility decline from 1960 to 2015. In other words, every 100 working age population in SSA would have to support 16 more children without the fertility declines that transpired in SSA countries. In Asia, the observed 2015 child dependency ratio was 36 and would have been 90 had there been no fertility decline. The fertility transition in the LAC region similarly reduced the child dependency ratio to 38, which would have been 92 had there been no fertility decline. The results are 52 vs. 102 in Northern Africa, meaning the fertility almost halved the burden of children on working age population.

**Figure 3.**
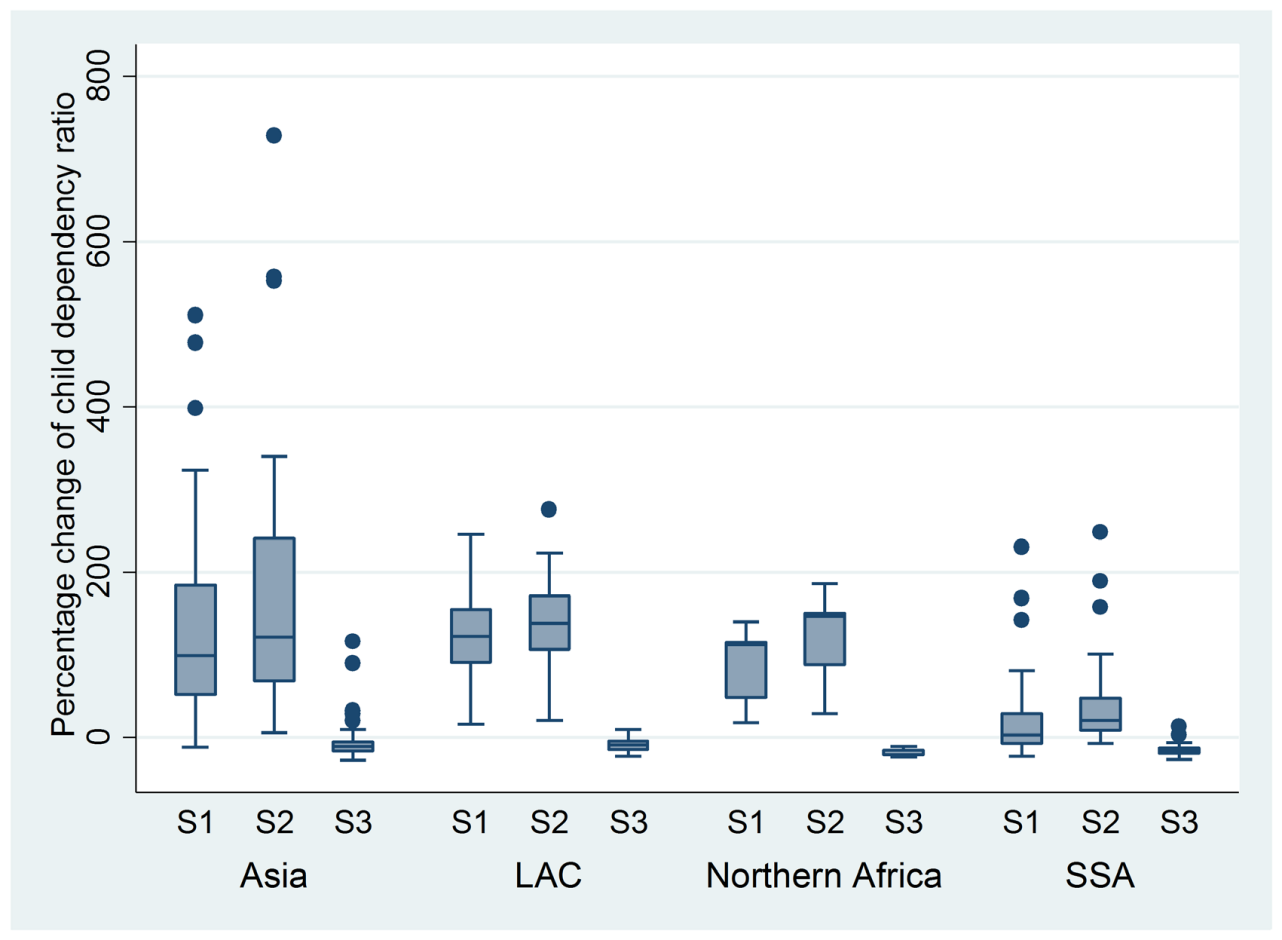
The percentage change in child dependency ratio from 1960 to 2015 in three fertility and mortality scenarios compared with UN data by regions. S1 = scenario 1, constant mortality and fertility; S2 = scenario 2, constant fertility; S3 = scenario 3, constant mortality.

These results are illustrated in Figure 3. We simulated how much higher the dependency ratios would be if mortality and/or fertility had been constant in the decades from 1960 to 2015. The percentage change in the child dependency ratio was positive and large in the constant fertility scenario in Asia and the LAC regions. The results underscore how notably fertility declines have reduced the child dependency ratio during the period 1960–2015. On the other hand, the percentage change in the child dependency ratio is negative for the constant mortality scenario, but the size of the change is marginal. The combined impact of mortality and fertility changes is positive for all of these five regions, but their size is substantially greater in Asia and LAC than in SSA.

**Figure 4.**
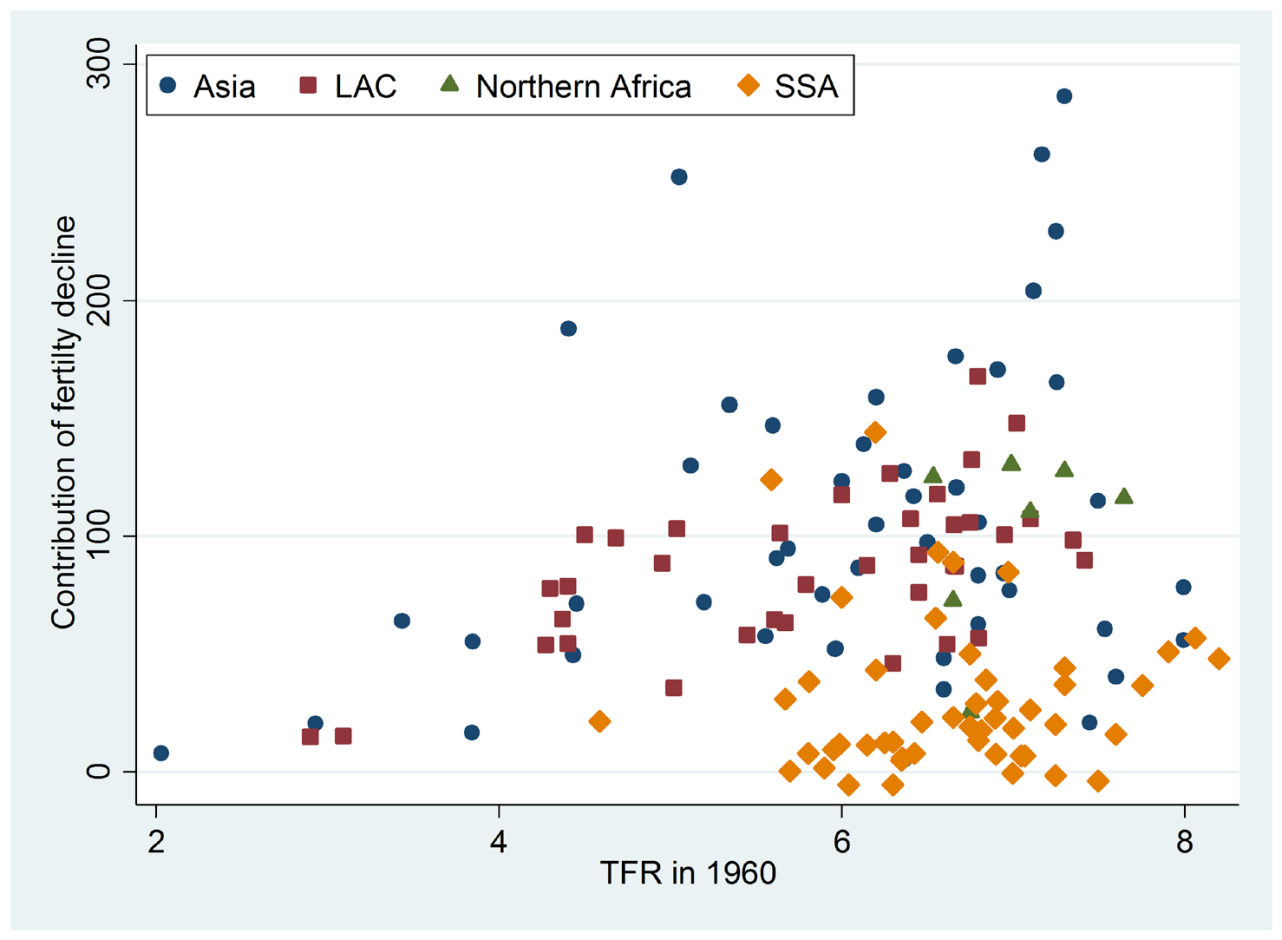
The relationship between total fertility rate in 1960 and the contribution of fertility decline to the change in total dependency ratio from 1960 to 2015.

Figure 4 shows a clear positive relationship between fertility levels in 1960 and the contribution of 1960–2015 fertility declines to 2015 total dependency ratios. This implies that previously high-fertility countries have catching up and they have a large potential to alter dependency ratio through fertility decline.

From the analyses conducted here, with the exception of most SSA and several Asian countries, a higher TFR in 1960 is associated with a larger contribution by fertility decline to the change in the child dependency ratio. The majority of high-fertility Asian countries have significantly reduced their TFR, resulting in smaller dependency ratios.

### Economic implications of fertility and mortality declines

There are several direct and indirect economic implications resulting from the changes in population age structure and consequences for the dependency ratio. By definition, GDP per capita can be broken down into GDP per worker and the proportion of working age population in the total population. By definition GDP per capita can be expressed as,

$$y_{it}=\frac{Y_{it}}{P_{it}}=\frac{Y_{it}}{W_{it}}\frac{W_{it}}{P_{it}}=z_{it}s_{it}\;(2)$$

where *Y _it_* is the gross domestic product (GDP) in country *i* in year *t*. *y _it_* is GDP per capita, *P _it_* is the total population, and *W _it_* is the number of workers. *z _it_* is the product per worker and *w _it_* the share of workers in the country at time *t*. In the present study, the number and proportion of workers are proxied by the working age population. Consequently, an increase in GDP per capita may be attributable to the change in either productivity per worker or the proportion of workers in the population.

This approach is a simplification of the actual change in GDP per capita, which can be affected by a variety of socio-economic, geographic, institutional and international factors ^15, 16^. The approach has been used in previous studies ^17^. An increased total dependency ratio means a reduced proportion of working age population, which, assuming a fixed worker productivity, indicates a lower GDP per capita. 

We simulated the GDP per capita that would have occurred had one factor not changed assuming that worker productivity did not change from 1960 to 2015. The gap between the actual and hypothetical values can be interpreted as the impact of the change in population age structure on GDP per capita. It is easy to show that,

$$y_{it}^{hat}=y_{it}^{obs}\frac{1+DR_{it}^{obs}/100}{1+DR_{it}^{hat}/100}\;(3)$$

where $y_{it}^{hat}$ denotes the GDP per capita in country *i* in year *t* had there been no fertility decline; $y_{it}^{obs}$ denotes the observed GDP per capita, i.e. from the World Bank database; $DR_{it}^{hat}$ and $DR_{it}^{obs}$ denote the total dependency ratio under those two scenarios.

As seen in the last three columns in Table 1 and Table 2, GDP would be much lower in most of the countries if the fertility decline between 1960 and 2015 had not occurred. Global GDP would decrease from the actual $106,422 billion to $87,406 billion without the fertility decline. The regional reductions are $12,390 billion in Asia, $1,706 billion in LAC, $484 billion in Northern Africa, and $321 billion in SSA. The estimated contributions are comparable to those of other studies. Bloom and Williamson (1998) and Bloom and Finlay (2009) suggested that the demographic transition accounted for between one fourth and two fifths of the “economic miracle” in East Asian Tigers’ economies ^1, 17^. A study projected that the demographic dividend could increase GDP per capita by about 11–32% in selected SSA countries over 2010–2040 under the UN’s low-fertility projection ^18^. Those estimates vary, mainly because they cover different periods in time. 

**Table 1.** The contribution of fertility and mortality decline to the change in dependency ratio (DR) and GDP per capita in 10.

Region	UN WPP 2017		Had there been no fertility change		Had there been no fertility and mortality change		GDP, PPP (constant 2011 international $)		
	Child DR	Total DR	Child DR	Total DR	Child DR	Total DR	World Bank	No fertility decline	Benefit of fertility decline
Asia (48)	36	47	90	77	792	575	49,969	37,579	12,390
Australia/New Zealand (2)	29	51	54	54	1,826	1,265	1,203	1,064	139
EUROPE (39)	24	50	41	39	2,193	1,812	23,073	21,367	1,706
LATIN AMERICA AND THE CARIBBEAN (29)	38	50	92	83	826	681	8,364	6,379	1,985
Melanesia, Micronesia, and Polynesia (7)	56	64	97	91	600	503	12	10	2
NORTHERN AMERICA (2)	28	51	53	52	1,691	1,279	18,434	16,446	1,989
Northern Africa (5)	52	61	102	86	658	613	2,035	1,551	484
Sub-Saharan Africa (46)	80	85	96	83	644	575	3,333	3,012	321
World (178)	40	52	85	73	937	759	106,422	87,406	19,016

**regions from 1960 to 2015** Note: the number of countries in each region is in the parenthesis.

**Table 2.** The contribution of fertility and mortality decline to the change in dependency ratio (DR) and GDP per capita in 201 countries from 1960 to 2015

Country	UN WPP			If no fertility decline			If no fertility or mortality decline			GDP ( constant 2011 international $)			
	Total DR	Child DR	Aged DR	Total DR	Child DR	Aged DR	Total DR	Child DR	Aged DR	GDP per capita	World Bank	No fertility decline	Benefit of fertility decline
Asia													
Afghanistan	89	84	5	21	22	4	-2	-1	-19	1,748	59	54	5
Armenia	44	29	16	71	136	-46	64	124	-46	8,180	24	20	4
Azerbaijan	40	32	8	123	160	-23	105	137	-20	16,699	161	119	42
Bahrain	30	27	3	262	282	79	221	241	44	44,456	61	38	23
Bangladesh	53	45	8	106	127	-19	71	90	-44	3,133	505	370	135
Bhutan	47	40	7	121	143	-9	78	97	-37	7,736	6	4	2
Brunei Darussalam	38	33	6	176	209	-12	165	199	-33	74,600	31	21	10
Cambodia	56	49	6	85	98	-16	68	82	-38	3,291	51	39	12
China	38	24	13	159	272	-49	120	226	-74	13,570	18,958	13,214	5,744
China, Hong Kong SAR	36	15	21	252	729	-98	129	398	-68	53,490	388	233	155
China, Macao SAR	27	16	11	188	304	20	166	295	-19	100,518	60	43	17
China, Taiwan Province of China	35	19	17	156	340	-51	141	323	-64	n/a	n/a	n/a	n/a
Cyprus	42	24	18	64	121	-12	54	115	-25	30,383	35	30	6
Dem. People's Republic of Korea	45	31	14	55	86	-12	41	76	-35	n/a	n/a	n/a	n/a
Georgia	50	28	22	20	57	-26	11	51	-39	9,025	36	33	2
India	52	44	9	75	94	-22	53	72	-43	5,754	7,532	5,988	1,544
Indonesia	49	42	8	90	110	-18	62	78	-23	10,368	2,677	2,062	614
Iran (Islamic Republic of)	40	33	7	171	212	-22	122	157	-44	16,010	1,271	853	418
Iraq	78	72	5	35	38	-8	20	22	-18	14,929	539	467	72
Israel	64	46	18	17	33	-23	9	30	-43	31,971	258	242	16
Japan	64	21	43	8	45	-11	-15	44	-44	37,818	4,840	4,698	142
Jordan	66	60	6	78	89	-26	64	75	-39	8,491	78	59	18
Kazakhstan	50	40	10	50	68	-23	39	54	-19	23,522	418	358	60
Kuwait	30	27	3	287	310	49	260	283	35	69,329	273	165	108
Kyrgyzstan	55	48	7	58	67	-10	45	52	-10	3,238	19	16	3
Lao People's Democratic Republic	60	54	6	52	59	-2	35	40	-15	5,434	36	30	6
Lebanon	47	35	12	95	141	-40	86	133	-55	13,087	77	59	18
Malaysia	45	36	8	127	165	-33	114	152	-46	24,989	768	551	217
Maldives	38	32	6	204	241	-3	129	156	-25	11,994	5	3	2
Mongolia	49	43	6	115	133	-23	86	102	-28	11,409	34	25	9
Myanmar	50	42	8	87	109	-31	64	84	-40	5,071	266	206	59
Nepal	61	53	9	52	65	-27	23	34	-44	2,301	66	55	11
Oman	32	29	3	230	243	103	173	184	65	40,139	169	108	61
Pakistan	65	58	7	48	56	-12	33	39	-13	4,695	889	746	143
Philippines	58	51	7	77	93	-35	67	82	-37	6,875	699	545	154
Qatar	18	16	1	541	558	325	493	511	268	119,749	297	164	133
Republic of Korea	37	19	18	147	332	-52	123	301	-68	34,178	1,729	1,239	490
Saudi Arabia	41	37	4	165	181	29	123	136	15	50,724	1,601	1,081	519
Singapore	37	21	16	130	260	-42	118	257	-67	80,892	448	331	117
Sri Lanka	51	37	14	72	115	-41	59	102	-54	11,062	229	184	45
State of Palestine	76	71	5	56	61	-13	41	46	-29	2,654	12	10	2
Syrian Arab Republic	73	66	7	61	71	-34	44	54	-46	n/a	n/a	n/a	n/a
Tajikistan	62	57	5	63	69	-2	45	50	1	2,641	23	18	4
Thailand	40	25	15	139	249	-49	117	220	-57	15,237	1,046	749	297
Timor-Leste	90	84	7	5	6	-1	-13	-12	-25	2,151	3	3	0
Turkey	50	38	12	105	148	-37	66	100	-47	23,382	1,830	1,355	475
Turkmenistan	53	46	6	83	97	-21	69	82	-26	14,992	83	65	19
United Arab Emirates	17	16	1	538	552	337	464	478	265	65,975	604	336	268
Uzbekistan	48	41	6	97	114	-14	84	99	-13	5,700	177	134	42
Viet Nam	43	33	10	117	158	-25	102	144	-43	5,667	530	393	137
Yemen	77	72	5	40	43	7	9	9	2	2,641	71	60	11
**Australia/New Zealand**													
Australia	51	28	23	38	82	-17	26	82	-44	43,832	1,043	925	119
New Zealand	53	31	22	43	102	-37	35	101	-54	34,646	160	139	21
**EUROPE**													
Albania	44	26	18	119	242	-58	102	211	-56	11,025	32	24	9
Austria	49	21	28	35	105	-17	20	101	-41	44,075	383	343	40
Belarus	44	23	21	26	72	-25	24	63	-20	17,230	163	151	12
Belgium	54	26	28	20	55	-14	5	53	-40	41,723	471	441	30
Bosnia and Herzegovina	43	21	23	62	174	-41	44	152	-55	10,902	39	32	6
Bulgaria	52	21	30	11	60	-24	7	53	-25	17,000	122	118	4
Channel Islands	47	22	25	37	78	2	24	77	-22	n/a	n/a	n/a	n/a
Croatia	51	22	28	14	50	-14	-2	46	-39	20,636	87	83	4
Czechia	50	23	27	19	49	-7	8	47	-25	30,381	322	304	19
Denmark	56	26	30	15	54	-20	6	52	-34	45,484	259	246	13
Estonia	54	25	29	7	17	-2	1	13	-10	27,329	36	35	1
Finland	58	26	32	13	60	-26	-1	60	-51	38,994	214	204	10
France	59	29	30	14	53	-23	1	51	-46	37,766	2,434	2,313	121
Germany	52	20	32	23	89	-19	7	85	-42	43,784	3,578	3,321	256
Greece	53	22	30	22	55	-1	3	51	-32	24,095	270	251	19
Hungary	47	21	26	15	26	6	7	18	-3	24,831	243	232	11
Iceland	52	31	21	48	102	-34	42	101	-46	42,674	14	12	2
Ireland	54	33	20	40	82	-28	32	80	-47	60,944	286	251	36
Italy	56	21	35	18	76	-18	1	72	-42	34,245	2,038	1,915	123
Latvia	52	23	29	6	22	-6	6	17	-2	23,057	46	45	1
Lithuania	50	22	28	18	71	-24	16	62	-20	26,971	79	75	4
Luxembourg	44	24	20	45	54	35	24	52	-9	95,311	54	47	7
Malta	49	21	27	35	126	-36	23	125	-57	34,380	15	13	2
Montenegro	48	27	21	40	93	-31	30	82	-39	15,291	10	9	1
Netherlands	53	26	27	29	90	-28	21	89	-42	46,354	785	714	72
Norway	52	27	25	29	65	-11	19	63	-29	63,670	331	301	30
Poland	44	21	22	37	99	-21	28	90	-32	25,299	968	869	99
Portugal	53	22	32	29	126	-36	13	107	-51	26,548	277	251	26
Republic of Moldova	35	21	13	71	128	-19	62	119	-28	4,747	19	16	3
Romania	48	23	25	15	38	-7	7	30	-13	20,538	408	390	18
Russian Federation	44	24	19	29	67	-18	27	57	-10	24,124	3,471	3,187	284
Serbia	49	25	24	18	55	-21	8	43	-28	13,278	118	111	7
Slovakia	42	22	20	49	114	-22	43	109	-28	28,254	154	134	19
Slovenia	49	22	27	26	63	-5	11	65	-33	29,097	60	56	5
Spain	51	23	29	31	87	-13	15	81	-38	32,216	1,495	1,352	143
Sweden	58	27	31	13	29	-1	1	28	-24	45,488	444	423	21
Switzerland	49	22	27	38	80	3	19	78	-29	56,511	470	418	52
TFYR Macedonia	42	24	18	66	138	-30	52	121	-40	12,760	27	22	4
Ukraine	45	22	23	19	51	-11	18	43	-6	7,465	333	315	18
United Kingdom	56	27	28	21	63	-20	9	62	-43	38,509	2,518	2,342	176
LATIN AMERICA AND THE CARIBBEAN													
Antigua and Barbuda	45	36	10	78	98	2	66	90	-21	20,114	2	2	0
Argentina	57	39	17	15	21	2	5	16	-19	19,101	829	786	43
Aruba	45	27	18	79	160	-46	73	155	-54	n/a	n/a	n/a	n/a
Bahamas	41	29	12	101	147	-15	88	137	-34	21,670	8	6	2
Barbados	50	29	21	54	132	-53	45	122	-60	15,390	4	4	1
Belize	57	51	6	92	106	-24	79	91	-24	8,061	3	2	1
Bolivia (Plurinational State of)	64	53	11	54	71	-32	31	47	-53	6,532	70	58	12
Brazil	44	32	11	117	172	-36	95	145	-48	14,666	3,021	2,224	796
Chile	45	30	15	89	150	-34	68	128	-51	22,537	400	314	87
Colombia	46	35	10	132	182	-40	112	160	-52	12,985	626	443	184
Costa Rica	45	32	13	127	194	-42	107	171	-55	14,914	72	51	20
Cuba	43	23	20	99	223	-46	85	206	-57	n/a	n/a	n/a	n/a
Curaçao	52	29	24	55	146	-56	48	141	-65	n/a	n/a	n/a	n/a
Dominican Republic	58	47	10	99	131	-50	73	102	-58	13,372	141	103	37
Ecuador	56	45	10	88	115	-32	66	91	-45	10,777	174	132	41
El Salvador	57	44	12	87	125	-48	60	94	-59	7,845	50	38	12
French Guiana	63	55	8	36	39	12	26	32	-20	n/a	n/a	n/a	n/a
Grenada	51	40	11	108	152	-55	94	136	-60	12,735	1	1	0
Guadeloupe	56	30	26	65	175	-66	55	168	-78	n/a	n/a	n/a	n/a
Guatemala	69	61	8	57	66	-20	35	44	-36	7,293	119	96	22
Guyana	54	46	8	88	110	-42	83	104	-41	7,063	5	4	1
Haiti	62	55	8	46	54	-9	27	34	-20	1,651	18	15	3
Honduras	60	53	7	90	105	-19	65	79	-40	4,311	39	29	10
Jamaica	49	35	14	101	160	-46	90	146	-53	8,105	23	17	6
Martinique	57	29	28	58	178	-66	48	171	-80	n/a	n/a	n/a	n/a
Mexico	51	42	10	106	139	-36	89	121	-45	16,668	2,098	1,543	555
Nicaragua	54	46	8	108	131	-30	81	103	-48	4,961	30	22	8
Panama	55	43	12	80	110	-31	67	98	-47	20,674	82	64	18
Paraguay	57	47	9	76	97	-29	68	90	-40	8,639	57	45	12
Peru	53	43	10	101	134	-37	71	101	-51	11,768	369	274	96
Puerto Rico	50	28	22	65	154	-49	58	146	-54	n/a	n/a	n/a	n/a
Saint Lucia	41	28	13	168	276	-59	146	246	-62	10,677	2	1	1
Saint Vincent and the Grenadines	47	36	11	148	209	-57	123	176	-55	10,463	1	1	0
Suriname	51	41	10	118	161	-56	108	150	-61	14,767	8	6	2
Trinidad and Tobago	43	30	13	103	173	-50	97	166	-57	31,283	43	32	10
United States Virgin Islands	61	33	28	63	181	-75	58	175	-81	n/a	n/a	n/a	n/a
Uruguay	56	33	23	15	34	-13	6	30	-29	19,831	68	65	3
Venezuela (Bolivarian Republic of)	53	43	10	105	137	-41	91	122	-51	n/a	n/a	n/a	n/a
Melanesia, Micronesia, and Polynesia													
Fiji	53	44	9	87	113	-43	75	100	-52	8,756	8	6	2
New Caledonia	48	34	14	76	124	-39	63	112	-53	n/a	n/a	n/a	n/a
Papua New Guinea	67	61	6	39	45	-16	29	36	-39	n/a	n/a	n/a	n/a
Solomon Islands	75	69	6	25	27	3	14	16	-12	2,053	1	1	0
Vanuatu	69	62	7	58	68	-29	40	49	-40	2,807	1	1	0
Guam	52	39	14	91	140	-49	80	130	-63	n/a	n/a	n/a	n/a
Kiribati	63	57	6	58	65	-16	46	53	-25	1,874	0	0	0
Micronesia (Fed. States of)	62	55	7	63	76	-33	56	68	-39	3,285	0	0	0
French Polynesia	45	35	11	108	153	-40	95	141	-58	n/a	n/a	n/a	n/a
Samoa	74	65	9	51	66	-54	43	60	-72	5,559	1	1	0
Tonga	74	64	10	38	52	-50	35	50	-62	5,189	1	0	0
NORTHERN AMERICA													
Canada	47	24	24	55	147	-36	45	144	-52	42,983	1,545	1,313	232
United States of America	51	29	22	34	80	-25	25	78	-44	52,790	16,889	15,132	1,757
Northern Africa													
Algeria	53	44	9	116	148	-38	84	113	-53	13,724	547	391	157
Egypt	62	54	8	73	89	-32	39	48	-21	10,096	947	741	206
Libya	49	43	6	128	151	-25	93	113	-38	n/a	n/a	n/a	n/a
Morocco	52	42	10	110	146	-46	83	115	-56	7,286	254	184	69
Sudan	82	75	6	26	29	-11	15	18	-19	4,290	166	149	17
Tunisia	46	34	11	130	186	-43	91	140	-59	10,750	121	86	35
Western Sahara	45	41	4	125	131	56	88	93	22	n/a	n/a	n/a	n/a
Sub-Saharan Africa													
Angola	98	93	5	16	17	1	-5	-4	-20	6,231	174	161	13
Benin	86	80	6	8	7	19	-9	-8	-12	1,987	21	20	1
Botswana	55	49	6	89	101	-16	68	79	-23	15,356	34	26	8
Burkina Faso	92	88	5	5	4	30	-13	-14	6	1,551	28	27	1
Burundi	90	85	5	7	6	21	-3	-4	6	749	8	7	0
Cabo Verde	55	48	7	85	102	-33	65	81	-46	5,919	3	2	1
Cameroon	86	80	6	8	7	15	-6	-7	4	2,991	68	66	2
Central African Republic	90	83	7	2	2	-9	-14	-14	-17	626	3	3	0
Chad	100	95	5	-5	-7	19	-17	-18	3	2,048	29	29	(1)
Comoros	76	70	5	30	32	-3	15	17	-16	1,413	1	1	0
Congo	84	78	6	12	12	4	2	2	-3	5,543	28	26	1
Côte d'Ivoire	84	78	5	37	41	-31	14	16	-21	3,251	75	64	11
Democratic Republic of the Congo	97	92	6	-5	-7	12	-16	-17	-1	750	57	59	(2)
Djibouti	57	50	6	65	72	14	49	55	2	3,139	3	2	1
Equatorial Guinea	68	63	5	31	29	50	10	9	22	27,238	32	28	4
Eritrea	85	78	7	18	20	-16	0	2	-18	n/a	n/a	n/a	n/a
Ethiopia	82	76	6	23	24	2	5	6	-16	1,533	153	139	14
Gabon	67	60	8	21	20	33	-1	-2	8	16,837	32	30	3
Gambia	92	88	4	0	-2	38	-21	-23	6	1,588	3	3	0
Ghana	73	67	6	39	43	-12	26	29	-18	3,930	108	93	15
Guinea	84	79	6	11	12	7	-8	-8	-16	1,184	14	14	1
Guinea-Bissau	80	75	5	9	9	20	-4	-4	5	1,424	3	2	0
Kenya	78	74	5	57	61	-16	38	42	-20	2,836	134	107	27
Lesotho	67	60	7	38	45	-15	21	27	-19	2,777	6	5	1
Liberia	83	78	6	21	23	2	-1	0	-23	785	4	3	0
Madagascar	80	75	5	37	40	-7	17	19	-21	1,376	33	29	5
Malawi	91	85	6	18	19	9	-7	-7	-5	1,089	19	18	2
Mali	102	97	5	-1	-2	16	-23	-23	-21	1,919	34	34	(0)
Mauritania	76	71	5	29	31	-1	17	19	-15	3,602	15	13	2
Mauritius	42	27	14	144	249	-60	130	231	-68	18,864	24	17	7
Mayotte	83	76	7	51	58	-34	40	48	-56	n/a	n/a	n/a	n/a
Mozambique	94	87	6	7	8	1	-11	-10	-18	1,118	31	30	1
Namibia	68	62	6	43	47	0	25	28	-11	9,913	24	20	4
Niger	112	106	5	-4	-4	2	-18	-17	-27	897	18	18	(0)
Nigeria	88	83	5	6	6	3	-8	-8	-4	5,671	1,027	999	28
Rwanda	77	72	5	48	51	-1	26	29	-16	1,716	20	16	3
Réunion	53	37	16	93	158	-58	78	142	-71	n/a	n/a	n/a	n/a
Sao Tome and Principe	87	81	6	13	14	-1	4	5	-14	2,942	1	1	0
Senegal	85	80	6	26	29	-8	5	8	-27	2,297	34	31	4
Seychelles	43	31	12	124	190	-50	108	169	-53	25,525	2	2	1
Sierra Leone	83	78	5	12	12	10	-11	-13	15	1,316	10	9	1
Somalia	97	92	5	-1	-2	12	-14	-14	-4	n/a	n/a	n/a	n/a
South Africa	53	45	8	74	92	-30	59	74	-29	12,425	687	547	140
South Sudan	84	77	6	19	21	2	-3	-1	-21	1,808	21	20	2
Swaziland	69	64	5	50	54	-3	30	34	-12	8,054	11	9	2
Togo	81	76	5	23	25	0	7	8	-7	1,351	10	9	1
Uganda	102	97	4	7	7	5	-6	-7	6	1,693	68	66	2
United Republic of Tanzania	93	87	6	13	15	-5	-3	-2	-15	2,491	134	126	8
Zambia	92	87	5	20	21	-5	5	5	-1	3,627	58	53	5
Zimbabwe	80	74	5	44	49	-19	28	31	-14	1,891	30	25	5

The estimation from this approach disregards the correlation between worker productivity and population age structure. Some studies have found that an increasing proportion of working age population is associated with improved worker productivity. Several mechanisms have been proposed to explain the association. As discussed above, an increased proportion of working age population, mostly brought about by rapid fertility decline, can be associated with an increased female labor participation rate ^9^. Declining fertility also encourages greater savings within the working age population for retirement ^14^. These behavioral changes promote the accumulation of financial and human capital, which will result in improved productivity per worker. Due to these associations between the proportions of working age population and worker productivity, the estimations of the impact of population age structure on GDP per capita presented here are conservative.

### Limitations

Although this paper has used widely recognized population data from the UN and World Bank and applied well-established demographic projection methods, it nevertheless is subject to limitations. Particularly, we assessed the changes in fertility, mortality, and dependency ratios for the decades from 1960 to 2015, a somewhat extended period of time during which many countries may have experienced short-term demographic and socioeconomic fluctuations. Our analysis cannot account for the impact of short-term variations on dependency ratios. As discussed above, the assumed independence of population size and age structure and worker productivity may be an oversimplification.

## Discussion

This study fills an important gap in the current literature on population welfare and reproductive and family health in LMICs. In PubMed we located about 500 articles published in English from Jan 1, 1990 to June 30, 2017 that included terms “fertility decline” or “mortality decline” in the titles or abstracts. But only 7 of them had “demographic dividend” in the titles or abstracts. Admittedly, many studies on the demographic dividend are published in the economic literature and thus may or may not be included in the PubMed database. However, our search results indicate that the demographic dividend perspective, which is appealing to policy makers, has not penetrated the health literature, and has not been fully utilized as evidence of the importance of improved reproductive, maternal and child health.

This study is the first to retrospectively assess the contribution of fertility and mortality declines to the change in national dependency ratios over the past five decades. It has also estimated the economic consequences of these demographic changes. Contrasting SSA to Asian and LAC countries sheds new light on the historical relationship between fertility, mortality and economic development. A favorable dependency ratio has enabled many Asian and LAC countries to realize the demographic dividend and to transform their predominantly rural agrarian economies into urban industrialized ones. During this period of development, many millions of people worldwide have been lifted out of poverty and their health substantially improved.

Assessing the contribution of fertility decline to the change in population age structure and GDP per capita provides a strong argument for expanding reproductive, maternal and child health interventions. Our study estimated the contribution of fertility declines, by far the more dominant factor, to the change in dependency ratios in 201 countries over the past five decades. Lower dependency ratios for countries as a whole as well as for individual households offer the opportunity to reallocate scarce resources toward better education, health care and nutrition. Improved health benefits for youth also confer stronger physical and cognitive performance with social and economic consequences that can disrupt poverty cycles.

The past half-century has been characterized by rapid demographic transitions and historically unprecedented economic growth in most parts of the world, with the exception of the SSA region. The population age structures in Asia and LAC experienced dramatic changes during the period 1960–2015. At the same time, countries in these regions were transformed from mostly rural agrarian economies with high fertility and mortality to largely urban industrialized ones with low fertility and mortality. In contrast, most SSA countries have lagged in their demographic transitions and economic development.

Based on a decomposition analysis of 201 countries, we found that fertility decline from 1960 to 2015 played a large role in changing the population age structure and lowering dependency ratios. Over this period, fertility decline contributed greatly to the reduction of the child dependency ratio in Asia and LAC while in contrast, its contribution in SSA was minor. The main reason is that fertility declined in SSA countries only marginally. The TFR in SSA fell from 6.67 in 1960 to 5.10 in 2015. During the same period, the TFR in LAC decreased from 5.89 to 2.14, and in Asia the change was from 5.81 to 2.20. The difference in the demographic transitions among these regions is consistent with the variation in their economic development.

Countries with slow fertility declines will need to accelerate the transitions in order to achieve a dependency ratio favorable for realizing a demographic dividend. Satisfying unmet need for family planning and providing full and voluntary access to a range of contraceptive methods have proven to be effective measures to reduce fertility. The implication of our study for policymakers is that expanding and intensifying the provision of effective reproductive, maternal, and child health interventions, particularly contraceptive access and nutrition enrichment, can accelerate ongoing fertility and mortality declines that contribute to population health as well as economic productivity and poverty alleviation. The induced benefits cover all three layers of the new paradigm of sustainable development - earth's life-support system, society, and economy. To ensure reaching the demographic dividends, governments of SSA countries should also encourage investments in human capital and ensure adequate employment, along with increased gender equity and nutrition ^19^.

## Data availability

All data used in the study are freely available online (no registration needed). Below are links to access the datasets:

- The 2017 Revision of World Population Prospects: https://esa.un.org/unpd/wpp/
- International Comparison Program Database of the World Bank: http://www.worldbank.org/en/programs/icp

## References

[1] Bloom DE Williamson JG: Demographic Transitions and Economic Miracles in Emerging Asia. *The World Bank Economic Review.* 1998;12(3):419–55. 10.1093/wber/12.3.419

[2] Caldwell JC: Toward A Restatement of Demographic Transition Theory. *Popul Dev Rev* 1976;2(3/4):321–66. 10.2307/1971615

[3] Griggs D Stafford-Smith M Gaffney O: Policy: Sustainable development goals for people and planet. *Nature.* 2013;495(7441):305–7. 10.1038/495305a 23518546

[4] Ahmed S Li Q Liu L: Maternal deaths averted by contraceptive use: an analysis of 172 countries. *Lancet.* 380(9837):111–25. 10.1016/S0140-6736(12)60478-4 22784531

[5] Malthus TR: An essay on the principle of population; or, A view of its past and present effects on human happiness. Reeves & Turner.1888 Reference Source

[6] Solow RM: A Contribution to the Theory of Economic Growth. *Q J Econ.* 1956;70(1):65–94. 10.2307/1884513

[7] Becker GS Glaeser EL Murphy KM: Population and Economic Growth. *Am Econ Rev.* 1999;89(2):145–9. 10.1257/aer.89.2.145

[8] Galor O Weil DN: Population, Technology, and Growth: From Malthusian Stagnation to the Demographic Transition and beyond. *Am Econ Rev.* 2000;90(4):806–28. 10.1257/aer.90.4.806

[9] Bloom DE Canning D Fink G: Fertility, Female Labor Force Participation, and the Demographic Dividend. *J Econ Growth.* 2009;14(2):79–101. 10.1007/s10887-009-9039-9

[10] Bloom DE Canning D Sevilla J: The Demographic Dividend A New Perspective on the Economic Consequences of Population Change. 1 ed : RAND Corporation.2003 10.7249/MR1274

[11] Brander JA Dowrick S: The role of fertility and population in economic growth: empirical research from aggregate cross-national data. *J Popul Econ.* 1994;7(1):1–25. 10.1007/BF00160435 12287546

[12] Crespo Cuaresma J Lutz W Sanderson W: Is the demographic dividend an education dividend? *Demography.* 2014;51(1):299–315. 10.1007/s13524-013-0245-x 24302530

[13] UN: World population prospects: the 2017 revision. Population division of the department of economic and social affairs of the United Nations Secretariat, New York.2017 Reference Source

[14] Lee R Mason A Miller T: Life Cycle Saving and the Demographic Transition: The Case of Taiwan. *Popul Dev Rev.* 2000;26:194–219. Reference Source

[15] Maddison A: A Comparison of Levels of GDP Per Capita in Developed and Developing Countries, 1700–1980. *J Econ Hist.* 1983;43(1):27–41. 10.1017/S0022050700028965

[16] Acemoglu D Johnson S Robinson JA: The Colonial Origins of Comparative Development: An Empirical Investigation. *Am Econ Rev.* 2001;91(5):1369–401. 10.1257/aer.91.5.1369

[17] Bloom DE Finlay JE: Demographic Change and Economic Growth in Asia. *Asian Econ Policy R.* 2009;4(1):45–64. 10.1111/j.1748-3131.2009.01106.x

[18] Mason A Lee R: Labor and Consumption across the Lifecycle. *J Econ Ageing.* 2013;1–2:16–27. 10.1016/j.jeoa.2013.06.002 24363989 PMC3866966

[19] Fabic MS Choi Y Bongaarts J: Meeting demand for family planning within a generation: the post-2015 agenda. *Lancet.* 2015;385(9981):1928–31. 10.1016/S0140-6736(14)61055-2 24993915 PMC4393371

