# Supplementary material for: Mortality, fertility, and economic development: An analysis of 201 countries from 1960 to 2015
Source: Gates Open Res. 2018 Mar 1;2:14. [Version 1] doi: 10.12688/gatesopenres.12804.1 (PMC5906751; doi:10.12688/gatesopenres.12804.1)
Supplement: Peer review history

# Peer review: Referee response for version 1

**DOI:** 10.21956/gatesopenres.13868.r26289

**Published:** 2018-04-09

**Authors:** Sri Moertiningsih Adioetomo, Qisha Quarina

**Affiliations:** 1 Faculty of Economics and Business, University of Indonesia, Depok, Indonesia

1. This article adds to the ‘not many’ available analyses that tried to explain the relationship between population growth and economic development (filling the gap). The availability of long-term population projection by age and trends in economic growth among developing countries made the analysis feasible. Congratulations.
2. Demographic Dividend is an emerging issue that tries to provide a solution or explanation on the long debate whether population growth has a negative or positive effect on economic growth. The work by economist demographers such as David Bloom, David Canning and Jaypee Sevilla (2003), other economists cited in Birdsall, Kelley and Sinding 2001 strongly favor the finding that it is the changes in age structure, that is the declining proportion of children accompanied by the rapid growth of population at the working age that boost economic growth.
3. The increase in the size of population at the working age 15-64 years decreases dependency ratio which boost economic growth through several assumptions: (1) the large size of workers will produce larger size of output which increases the national GDP; (2) this will promote savings (capital accumulation); (3) the decreasing family size (due to family planning) eases women to participate in the labor force which in turn helps increase GDP growth; (4) but the most important thing is policy on human capital development (Bloom *et.al.* 2003).
4. There is also an issue about the impact of the very rapid fertility decline and the demographic transition in developing countries due to strong family planning program, such as in China, Thailand, and Indonesia. In the Western countries, the process of demographic transition occurred slowly and took more than one hundred years and accompanied by social and economic development. While in these developing countries such as Indonesia, demographic transition took only in three decades, when social and economic development in these countries have just begun. The population at the working age are not ready to utilize the favorable age structure for economic growth due to the low quality of workers.
5. Economist and policy makers (especially in Indonesia) argue that demographic dividend is not automatically being harnessed. To be able to boost economic growth appropriate policy measures, especially investment on human capital is the key to reap the demographic dividend.
6. Nevertheless this article is well appreciated, it adds to the existing literature which tried to provide the explanation on the link between population growth and economic growth. This article is powerful as advocacy tool for policy makers, that family planning program is not a cost, it is an investment.
7. But this analysis can be well improved through the inclusion of other variables measuring the quality of human capital of the related countries.

We have read this submission. We believe that we have an appropriate level of expertise to confirm that it is of an acceptable scientific standard.

# Peer review: Referee response for version 1

**DOI:** 10.21956/gatesopenres.13868.r26288

**Published:** 2018-03-13

**Authors:** Usha Ram

**Affiliations:** 1 Department of Public Health and Mortality Studies, International Institute for Population Sciences, Mumbai, India

My general observations on the paper are as below:

 The paper very clearly demonstrates economic advantages through empowering women as a result of fertility reduction over a period of 55 years. In the past, many researchers have attempted to demonstrate the same by projecting population based on various assumption on future fertility regime. However, the present research is different in the sense it compares hypothetical, yet, simple assumptions of constant fertility and constant fertility and mortality and simulates 2015 situation with actual experiences for 2015 in 201 countries covering different regions.

 The present work clearly advocates that if the countries and global communities had not invested in the family planning, the world and each country could have been unmanageably very different and possibly it would have been difficult to achieve the current wellbeing levels of the populations and especially the lives of the women who had been benefited the most from these investments. It might have sometimes difficult for some countries when they adopted certain path which were the violation of human right but corrective steps by mechanism of Global pressures and guidelines for changing the course of program focus. In the past few decades, there has been a feeling around the globe that the population problems have largely been solved with the exception of a few geographies.

 The paper again demonstrates incomplete agenda of reproductive rights of women and advocates more economic returns than the investment required to be made in the family planning program. The value of research is enhanced in view of London Submit of 2012 that expressed its commitment to reach 120 million unreached women out there needing family planning services. Even country like India that has already reached TFR of 2.2 per women in 2015 has more than 30 million unmet need for contraception (if we express demand for modern method then this number would be close to 45 million).

 This also means that our demographic goal may be replacement fertility but demand for contraception in any country would go beyond this and would depend on method-mix, access to basket of methods. In this context, I strongly believe that such research work provides program managers and policy makers of country to rethink about the invest strategies for empowering women. It will be observed then that this option would be easier and cheaper than any other option available to empower women. I also believe that such analysis would be very useful at state level for a country like India that has huge demographic diversity.

I have read this submission. I believe that I have an appropriate level of expertise to confirm that it is of an acceptable scientific standard.

# Peer review: Referee response for version 1

**DOI:** 10.21956/gatesopenres.13868.r26290

**Published:** 2018-03-12

**Authors:** John May, Vincent Turbat

**Affiliations:** 1 Georgetown University, Washington, DC, USA

First, we would suggest that the paper focuses on the main topic, namely the relationship between mortality and fertility, on the one hand, and the demographic dependency ratio and economic development (the demographic dividend), on the other. Therefore, the discussion on Malthus (see Introduction) is not needed and only sidetracks the reader.

 Second, the demographic dependency ratio as used in the paper is rather narrow with respect to young dependents (age group 0 to 14). We would suggest the authors use a broader age group for the young dependents, namely age group 0 to 19 completed (or age group under age 20 exact). So the entire paper might use this extended group of young dependents. In this respect, see John F. May and Vincent Turbat, “Demographic Dividend in Sub-Saharan Africa: Two Issues that Need More Attention”, Journal of Demographic Economics, vol. 83(1), March 2017, pp. 77-84. ^1^

 Third, some definitions used in the paper need to be refined or expanded. The demographic dividend is a process leading to an economic surplus; it should not be presented as a result. Likewise, the definition of productivity used in the paper is weak. We believe the authors meant the average production per capita.

 Fourth, we believe the authors used the 2017 UN Population Division projections; however, this is not clearly explained in the paper and sometimes the reader is under the impressions that the authors used their own population projections (see section on CCM) page 5). In addition, the economic model used in the paper needs more explanations and also double-checking. For instance, the explanation of equation (2) page 12 should cite s(it) and not w(it) (see 3 ^rd^ line below equation (2).

 Fifth, we have some problems with Table 1, first line pertaining to Asia. The Child DR with no fertility change is 90, but the Total DR with no fertility change is 77; how can the latter figure be lower than the first? In addition, the Child DR and Total DR with no fertility and mortality change jump to 792 and 575, respectively. How is this possible, especially as the authors explain that fertility is a more important factor than fertility to explain changes in the DR? We were puzzled.

 Finally, the style of the paper needs to be improved. It seems that the paper was written rather rapidly. In addition, Table 2 (which is very long) could be presented as an Annex.

We have read this submission. We believe that we have an appropriate level of expertise to confirm that it is of an acceptable scientific standard, however we have significant reservations, as outlined above.

## References

[1] MayJTurbatV: THE DEMOGRAPHIC DIVIDEND IN SUB-SAHARAN AFRICA: TWO ISSUES THAT NEED MORE ATTENTION. *Journal of Demographic Economics*.2017;83(01) : 10.1017/dem.2016.21 77-84 10.1017/dem.2016.21
